# Dibromido(6-methyl-2,2′-bipyridine-κ^2^ *N*,*N*′)cobalt(II)

**DOI:** 10.1107/S1600536812045230

**Published:** 2012-11-07

**Authors:** Sadif A. Shirvan, Sara Haydari Dezfuli, Fereydoon Khazali, Ali Borsalani

**Affiliations:** a Department of Chemistry, Omidieh Branch, Islamic Azad University, Omidieh, Iran; b Department of Petroleum Engineering, Omidieh Branch, Islamic Azad University, Omidieh, Iran

## Abstract

In the mol­ecule of the title compound, [CoBr_2_(C_11_H_10_N_2_)], the Co^II^ atom is four-coordinated in a distorted tetra­hedral geometry by two N atoms from a chelating 6-methyl-2,2′-bipyridine ligand and two terminal Br atoms. In the crystal, π–π stacking inter­actions between the pyridine rings along the *a*-axis direction [centroid–centroid distance = 3.761 (7) Å] and C—H⋯Br hydrogen bonds in the *bc* plane together generate the three-dimensional packing.

## Related literature

For related structures, see: Ahmadi *et al.* (2008*a* ▶,*b* ▶, 2009 ▶); Amani *et al.* (2009 ▶); Kalateh *et al.* (2010 ▶); Newkome *et al.* (1982 ▶); Onggo *et al.* (2005 ▶); Shirvan *et al.* (2012 ▶); Shirvan & Haydari Dezfuli (2012 ▶).
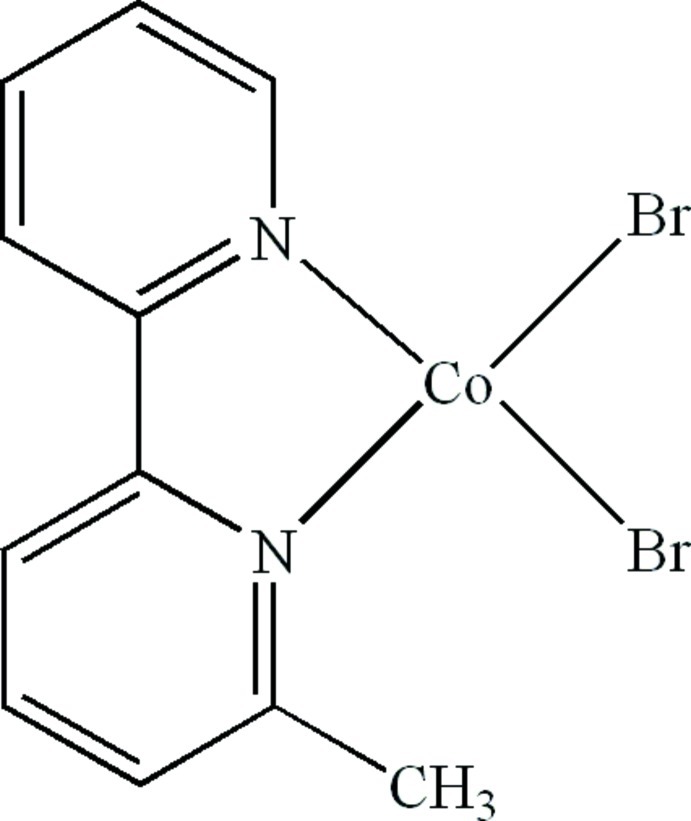


## Experimental

### 

#### Crystal data

[CoBr_2_(C_11_H_10_N_2_)]
*M* *_r_* = 388.94
Monoclinic, 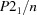

*a* = 7.5541 (7) Å
*b* = 9.7249 (7) Å
*c* = 17.7352 (16) Å
β = 97.392 (7)°
*V* = 1292.05 (19) Å^3^
*Z* = 4
Mo *K*α radiation
μ = 7.49 mm^−1^
*T* = 173 K
0.45 × 0.13 × 0.10 mm

#### Data collection

Bruker APEXII CCD diffractometer
Absorption correction: multi-scan (*SADABS*; Bruker, 2001 ▶) *T*_min_ = 0.379, *T*_max_ = 0.512
6393 measured reflections
2519 independent reflections
1546 reflections with *I* > 2σ(*I*)
*R*_int_ = 0.099

#### Refinement

*R*[*F* ^2^ > 2σ(*F* ^2^)] = 0.085
*wR*(*F* ^2^) = 0.164
*S* = 1.05
2519 reflections
145 parameters
H-atom parameters constrained
Δρ_max_ = 2.16 e Å^−3^
Δρ_min_ = −1.13 e Å^−3^

### 

Data collection: *APEX2* (Bruker, 2007 ▶); cell refinement: *SAINT* (Bruker, 2007 ▶); data reduction: *SAINT*; program(s) used to solve structure: *SHELXS97* (Sheldrick, 2008 ▶); program(s) used to refine structure: *SHELXL97* (Sheldrick, 2008 ▶); molecular graphics: *ORTEP-3* (Farrugia, 1997 ▶) and *Mercury* (Macrae *et al.*, 2006 ▶); software used to prepare material for publication: *SHELXL97*.

## Supplementary Material

Crystal structure: contains datablock(s) I, global. DOI: 10.1107/S1600536812045230/hy2600sup1.cif

Structure factors: contains datablock(s) I. DOI: 10.1107/S1600536812045230/hy2600Isup2.hkl

Additional supplementary materials:  crystallographic information; 3D view; checkCIF report

## Figures and Tables

**Table 1:** Selected bond lengths (Å)

Co1—Br1	2.352 (2)
Co1—Br2	2.3698 (19)
Co1—N1	2.035 (10)
Co1—N2	2.029 (8)

**Table 2:** Hydrogen-bond geometry (Å, °)

*D*—H⋯*A*	*D*—H	H⋯*A*	*D*⋯*A*	*D*—H⋯*A*
C1—H1*A*⋯Br1^i^	0.96	2.89	3.849 (14)	178
C8—H8⋯Br2^ii^	0.93	2.89	3.771 (14)	158

Symmetry codes: (i) 
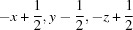
; (ii) 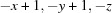.

**Fig. 1.**
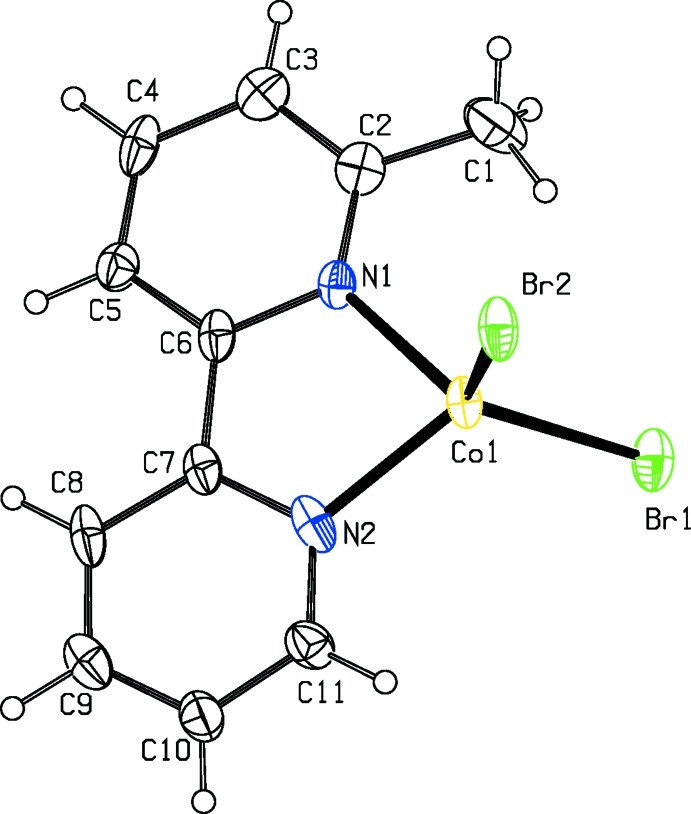
The molecular structure of the title compound. Displacement ellipsoids are drawn at the 30% probability level.

**Fig. 2.**
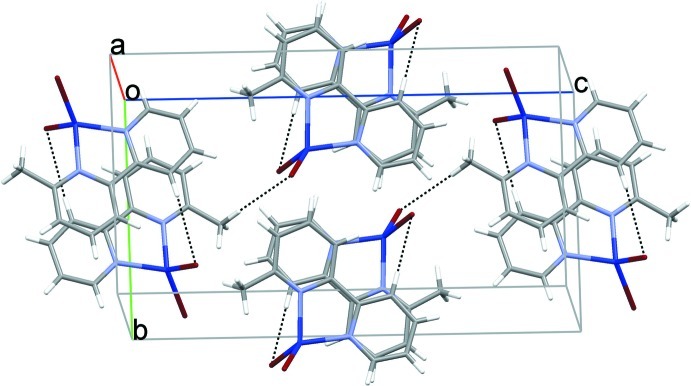
Crystal packing diagram for the title compound. Hydrogen bonds are shown as dashed lines.

## supplementary crystallographic information

### Comment

Recently, we reported the synthesis and crystal structure of [In(6-mbipy)Cl_3_(DMSO)], (II) (Shirvan *et al.*, 2012) and [Cd(6-mbipy)Br_2_(DMSO)], (III) (Shirvan & Haydari Dezfuli, 2012) (6-mbipy = 6-methyl-2,2'-bipyridine, DMSO = dimethyl sulfoxide). 6-Methyl-2,2'-bipyridine is a good ligand and a few complexes with 6-mbipy have been prepared, such as that of [Hg(6-mbipy)Cl_2_], (IV) (Ahmadi *et al.*, 2008*a*), [Pt(6-mbipy)Cl_4_], (V) (Amani *et al.*, 2009), [Pb_4_(NO_3_)_8_(6-mbipy)_4_], (VI) (Ahmadi *et al.*, 2009), [Zn(6-mbipy)Br_2_], (VII) (Kalateh *et al.*, 2010), [Zn(6-mbipy)Cl_2_], (VIII) (Ahmadi *et al.*, 2008*b*), [Pd(6-mbipy)Cl_2_], (IX) (Newkome *et al.*, 1982) and [Ru(6-mbipy)_3_][BF_4_]_2_, (X) (Onggo *et al.*, 2005). We report herein the synthesis and crystal structure of the title compound, (I).

In the title compound (Fig. 1), the Co^II^ atom is four-coordinated in a distorted tetrahedral geometry by two N atoms from a chelating 6-mbipy ligand and two terminal Br atoms (Table 1). In the crystal, intermolecular C—H···Br hydrogen bonds and π–π contacts (Table 2, Fig. 2) between the pyridine rings, *Cg*2···*Cg*3^i^ [symmetry code: (i) 1-x, 1-y, -z, *Cg*2 and *Cg*3 are the centroids of the rings N1/C2–C6 and N2/C7–C11, respectively], with a centroid–centroid distance of 3.761 (7) Å, stabilize the structure.

### Experimental

For the preparation of the title compound, a solution of 6-mbipy (0.28 g, 0.26 ml, 1.65 mmol) in acetonitrile (10 ml) was added to a solution of CoBr_2_ (0.36 g, 1.65 mmol) in acetonitrile (10 ml) and the resulting blue solution was stirred for 20 min at 313 K. This solution was left to evaporate slowly at room temperature. After one week, blue needle crystals of the title compound were isolated (yield: 0.47 g, 73.2%).

### Refinement

All H atoms were positioned geometrically and refined as riding atoms, with C—H = 0.93 (CH) and 0.96 (CH_3_) Å and with *U*_iso_(H) = 1.2*U*_eq_(C). The highest residual electron density was found 0.96 Å from Br1 the deepest hole 0.79 Å from Br1.

### Crystal data

**Table:**

[CoBr_2_(C_11_H_10_N_2_)]	*F*(000) = 748
*M**_r_* = 388.94	*D*_x_ = 2.000 Mg m^−^^3^
Monoclinic, *P*2_1_/*n*	Mo *K*α radiation, λ = 0.71073 Å
Hall symbol: -P 2yn	Cell parameters from 6393 reflections
*a* = 7.5541 (7) Å	θ = 3.1–26.0°
*b* = 9.7249 (7) Å	µ = 7.49 mm^−^^1^
*c* = 17.7352 (16) Å	*T* = 173 K
β = 97.392 (7)°	Needle, blue
*V* = 1292.05 (19) Å^3^	0.45 × 0.13 × 0.10 mm
*Z* = 4	

### Data collection

**Table:**

Bruker APEXII CCD diffractometer	2519 independent reflections
Radiation source: fine-focus sealed tube	1546 reflections with *I* > 2σ(*I*)
Graphite monochromator	*R*_int_ = 0.099
φ and ω scans	θ_max_ = 26.0°, θ_min_ = 3.1°
Absorption correction: multi-scan (*SADABS*; Bruker, 2001)	*h* = −7→9
*T*_min_ = 0.379, *T*_max_ = 0.512	*k* = −10→11
6393 measured reflections	*l* = −21→21

### Refinement

**Table:**

Refinement on *F*^2^	Primary atom site location: structure-invariant direct methods
Least-squares matrix: full	Secondary atom site location: difference Fourier map
*R*[*F*^2^ > 2σ(*F*^2^)] = 0.085	Hydrogen site location: inferred from neighbouring sites
*wR*(*F*^2^) = 0.164	H-atom parameters constrained
*S* = 1.05	*w* = 1/[σ^2^(*F*_o_^2^) + (0.0569*P*)^2^ + 5.329*P*] where *P* = (*F*_o_^2^ + 2*F*_c_^2^)/3
2519 reflections	(Δ/σ)_max_ = 0.002
145 parameters	Δρ_max_ = 2.16 e Å^−^^3^
0 restraints	Δρ_min_ = −1.13 e Å^−^^3^

### Special details

**Table:**

Geometry. All e.s.d.'s (except the e.s.d. in the dihedral angle between two l.s. planes) are estimated using the full covariance matrix. The cell e.s.d.'s are taken into account individually in the estimation of e.s.d.'s in distances, angles and torsion angles; correlations between e.s.d.'s in cell parameters are only used when they are defined by crystal symmetry. An approximate (isotropic) treatment of cell e.s.d.'s is used for estimating e.s.d.'s involving l.s. planes.
Refinement. Refinement of *F*^2^ against ALL reflections. The weighted *R*-factor *wR* and goodness of fit *S* are based on *F*^2^, conventional *R*-factors *R* are based on *F*, with *F* set to zero for negative *F*^2^. The threshold expression of *F*^2^ > σ(*F*^2^) is used only for calculating *R*-factors(gt) *etc*. and is not relevant to the choice of reflections for refinement. *R*-factors based on *F*^2^ are statistically about twice as large as those based on *F*, and *R*- factors based on ALL data will be even larger.

### Fractional atomic coordinates and isotropic or equivalent isotropic displacement parameters (Å^2^)

**Table:**

	*x*	*y*	*z*	*U*_iso_*/*U*_eq_	
C3	0.1467 (17)	0.3377 (14)	0.1282 (7)	0.046 (3)	
H3	0.1126	0.2832	0.1669	0.056*	
C4	0.1737 (16)	0.2801 (14)	0.0583 (7)	0.046 (3)	
H4	0.1588	0.1861	0.0501	0.055*	
Br1	0.04264 (18)	0.91295 (15)	0.11995 (7)	0.0501 (4)	
N2	0.2940 (12)	0.7342 (12)	−0.0213 (5)	0.042 (3)	
C6	0.2426 (15)	0.5016 (13)	0.0149 (6)	0.034 (3)	
C7	0.2897 (14)	0.6000 (13)	−0.0435 (6)	0.034 (3)	
C8	0.3225 (16)	0.5617 (15)	−0.1157 (6)	0.045 (3)	
H8	0.3190	0.4698	−0.1303	0.054*	
C9	0.3607 (16)	0.6643 (16)	−0.1658 (6)	0.045 (4)	
H9	0.3865	0.6417	−0.2142	0.055*	
C10	0.3601 (18)	0.7992 (16)	−0.1431 (6)	0.049 (4)	
H10	0.3798	0.8692	−0.1768	0.059*	
N1	0.2173 (12)	0.5578 (10)	0.0835 (5)	0.031 (2)	
C11	0.3303 (17)	0.8297 (15)	−0.0711 (6)	0.043 (3)	
H11	0.3356	0.9212	−0.0558	0.052*	
Br2	0.54925 (17)	0.79979 (15)	0.16326 (7)	0.0467 (4)	
Co1	0.2680 (2)	0.76329 (17)	0.09004 (8)	0.0345 (4)	
C5	0.2222 (17)	0.3629 (13)	0.0021 (6)	0.040 (3)	
H5	0.2414	0.3255	−0.0444	0.047*	
C2	0.1726 (16)	0.4806 (14)	0.1384 (7)	0.041 (3)	
C1	0.144 (2)	0.5546 (17)	0.2094 (7)	0.070 (5)	
H1A	0.2213	0.5171	0.2516	0.084*	
H1B	0.0218	0.5439	0.2183	0.084*	
H1C	0.1697	0.6505	0.2042	0.084*	

### Atomic displacement parameters (Å^2^)

**Table:**

	*U*^11^	*U*^22^	*U*^33^	*U*^12^	*U*^13^	*U*^23^
C3	0.033 (7)	0.049 (9)	0.057 (7)	0.002 (7)	0.002 (6)	0.001 (6)
C4	0.036 (7)	0.036 (8)	0.061 (8)	0.007 (6)	−0.010 (6)	−0.022 (6)
Br1	0.0389 (8)	0.0543 (10)	0.0579 (8)	0.0073 (7)	0.0089 (6)	−0.0186 (6)
N2	0.023 (5)	0.072 (8)	0.029 (5)	0.002 (5)	0.002 (4)	−0.013 (5)
C6	0.020 (6)	0.042 (7)	0.038 (6)	0.008 (6)	−0.002 (4)	−0.013 (5)
C7	0.020 (6)	0.047 (8)	0.033 (6)	0.007 (6)	0.002 (4)	−0.009 (5)
C8	0.034 (7)	0.062 (9)	0.037 (6)	0.010 (7)	−0.002 (5)	−0.026 (6)
C9	0.025 (6)	0.077 (11)	0.035 (6)	0.010 (7)	0.005 (5)	−0.005 (6)
C10	0.053 (9)	0.060 (10)	0.035 (6)	0.019 (8)	0.005 (6)	−0.003 (6)
N1	0.021 (5)	0.034 (6)	0.039 (5)	−0.002 (4)	0.010 (4)	−0.007 (4)
C11	0.046 (8)	0.047 (9)	0.038 (6)	−0.012 (7)	0.011 (5)	−0.001 (6)
Br2	0.0342 (7)	0.0557 (9)	0.0488 (7)	0.0003 (7)	−0.0004 (5)	−0.0266 (6)
Co1	0.0306 (9)	0.0390 (11)	0.0347 (8)	−0.0018 (8)	0.0070 (6)	−0.0138 (7)
C5	0.042 (8)	0.034 (7)	0.042 (6)	0.001 (6)	0.002 (5)	−0.006 (6)
C2	0.026 (7)	0.046 (8)	0.049 (7)	−0.005 (6)	−0.001 (5)	−0.005 (6)
C1	0.095 (13)	0.077 (12)	0.040 (7)	−0.029 (10)	0.014 (7)	−0.001 (7)

### Geometric parameters (Å, º)

**Table:**

Co1—Br1	2.352 (2)	C7—C8	1.386 (14)
Co1—Br2	2.3698 (19)	C8—C9	1.391 (19)
Co1—N1	2.035 (10)	C8—H8	0.9300
Co1—N2	2.029 (8)	C9—C10	1.372 (19)
C3—C4	1.399 (17)	C9—H9	0.9300
C3—C2	1.411 (18)	C10—C11	1.357 (16)
C3—H3	0.9300	C10—H10	0.9300
C4—C5	1.366 (18)	N1—C2	1.309 (15)
C4—H4	0.9300	C11—H11	0.9300
N2—C11	1.334 (16)	C5—H5	0.9300
N2—C7	1.362 (16)	C2—C1	1.491 (18)
C6—N1	1.369 (13)	C1—H1A	0.9600
C6—C5	1.373 (17)	C1—H1B	0.9600
C6—C7	1.487 (17)	C1—H1C	0.9600
			
C4—C3—C2	118.3 (13)	C2—N1—C6	120.7 (11)
C4—C3—H3	120.8	C2—N1—Co1	125.8 (8)
C2—C3—H3	120.8	C6—N1—Co1	113.3 (8)
C5—C4—C3	119.5 (13)	N2—C11—C10	123.0 (13)
C5—C4—H4	120.2	N2—C11—H11	118.5
C3—C4—H4	120.2	C10—C11—H11	118.5
C11—N2—C7	118.3 (10)	N2—Co1—N1	81.3 (4)
C11—N2—Co1	127.0 (9)	N2—Co1—Br1	118.0 (3)
C7—N2—Co1	114.4 (8)	N1—Co1—Br1	119.0 (3)
N1—C6—C5	120.9 (11)	N2—Co1—Br2	111.2 (3)
N1—C6—C7	115.8 (10)	N1—Co1—Br2	109.1 (3)
C5—C6—C7	123.3 (10)	Br1—Co1—Br2	114.06 (7)
N2—C7—C8	121.6 (12)	C4—C5—C6	119.6 (11)
N2—C7—C6	114.5 (9)	C4—C5—H5	120.2
C8—C7—C6	123.9 (12)	C6—C5—H5	120.2
C7—C8—C9	118.3 (13)	N1—C2—C3	120.9 (11)
C7—C8—H8	120.9	N1—C2—C1	115.6 (12)
C9—C8—H8	120.9	C3—C2—C1	123.4 (12)
C10—C9—C8	119.3 (11)	C2—C1—H1A	109.5
C10—C9—H9	120.3	C2—C1—H1B	109.5
C8—C9—H9	120.3	H1A—C1—H1B	109.5
C11—C10—C9	119.5 (13)	C2—C1—H1C	109.5
C11—C10—H10	120.3	H1A—C1—H1C	109.5
C9—C10—H10	120.3	H1B—C1—H1C	109.5
			
C2—C3—C4—C5	−0.6 (18)	C7—N2—Co1—N1	−7.8 (8)
C11—N2—C7—C8	0.2 (16)	C11—N2—Co1—Br1	60.6 (11)
Co1—N2—C7—C8	−173.8 (8)	C7—N2—Co1—Br1	−126.1 (7)
C11—N2—C7—C6	−178.2 (10)	C11—N2—Co1—Br2	−74.0 (10)
Co1—N2—C7—C6	7.8 (12)	C7—N2—Co1—Br2	99.4 (7)
N1—C6—C7—N2	−2.6 (14)	C2—N1—Co1—N2	−176.7 (10)
C5—C6—C7—N2	176.5 (11)	C6—N1—Co1—N2	6.3 (7)
N1—C6—C7—C8	179.1 (10)	C2—N1—Co1—Br1	−59.5 (10)
C5—C6—C7—C8	−1.8 (18)	C6—N1—Co1—Br1	123.5 (7)
N2—C7—C8—C9	0.1 (17)	C2—N1—Co1—Br2	73.7 (9)
C6—C7—C8—C9	178.3 (11)	C6—N1—Co1—Br2	−103.3 (7)
C7—C8—C9—C10	−1.7 (18)	C3—C4—C5—C6	−0.5 (18)
C8—C9—C10—C11	3.1 (19)	N1—C6—C5—C4	0.9 (18)
C5—C6—N1—C2	−0.2 (16)	C7—C6—C5—C4	−178.1 (10)
C7—C6—N1—C2	178.9 (10)	C6—N1—C2—C3	−0.9 (17)
C5—C6—N1—Co1	177.0 (9)	Co1—N1—C2—C3	−177.7 (9)
C7—C6—N1—Co1	−3.9 (11)	C6—N1—C2—C1	−178.3 (11)
C7—N2—C11—C10	1.2 (18)	Co1—N1—C2—C1	4.9 (15)
Co1—N2—C11—C10	174.3 (10)	C4—C3—C2—N1	1.3 (18)
C9—C10—C11—N2	−3 (2)	C4—C3—C2—C1	178.5 (13)
C11—N2—Co1—N1	178.8 (11)		

### Hydrogen-bond geometry (Å, º)

**Table:**

*D*—H···*A*	*D*—H	H···*A*	*D*···*A*	*D*—H···*A*
C1—H1*A*···Br1^i^	0.96	2.89	3.849 (14)	178
C8—H8···Br2^ii^	0.93	2.89	3.771 (14)	158

Symmetry codes: (i) −*x*+1/2, *y*−1/2, −*z*+1/2; (ii) −*x*+1, −*y*+1, −*z*.
